# Genome Sequences of Newly Emerged Newcastle Disease Virus Strains Isolated from Disease Outbreaks in Indonesia

**DOI:** 10.1128/MRA.00204-20

**Published:** 2020-06-04

**Authors:** Mohammad Rabiei, Mohamad Indro Cahyono, Phuong Thi Kim Doan, Putri Pandarangga, Simson Tarigan, Risa Indriani, Indi Dharmayanti, Jagoda Ignjatovic, Wai Yee Low, Rick Tearle, Milton M. McAllister, Mohammed Alsharifi, Farhid Hemmatzadeh

**Affiliations:** a School of Animal and Veterinary Sciences, The University of Adelaide, Adelaide, Australia; b Indonesian Research Centre for Veterinary Science, Bogor, West Java, Indonesia; c School of Animal and Veterinary Sciences, Tay Nguyen University, Dak Lak, Vietnam; d Department of Veterinary Pathology, Nusa Cendana University, Kupang, Indonesia; e School of Veterinary Science, The University of Melbourne, Melbourne, Victoria, Australia; f Davies Research Centre, School of Animal and Veterinary Sciences, The University of Adelaide, Adelaide, Australia; g Research Centre for Infectious Diseases, School of Biological Sciences, University of Adelaide, Adelaide, Australia; DOE Joint Genome Institute

## Abstract

Here, we report two genomes of newly emerged strains of *Newcastle disease virus* (NDV), Chicken/Indonesia/Tangerang/004WJ/14 and Chicken/Indonesia/VD/003WJ/11, from disease outbreaks in chickens in Indonesia. Phylogenetic study results of the fusion (F) protein’s gene-coding sequences of different genotypes of NDV revealed that these two strains belong to genotype VII.2 in the class II cluster of avian paramyxoviruses.

## ANNOUNCEMENT

Newcastle disease (ND) still causes high mortality and reduces profitability in the chicken industry in Southeast Asia and is an endemic disease in Indonesia. Newcastle disease virus (NDV) is a member of the genus *Avian orthoavulavirus 1* within the new subfamily *Avulavirinae* of the family *Paramyxoviridae* (1). Genotypes VII.1.1 (subgenotypes b, d, e, j, and l) and VII.2 (subgenotypes a, h, i, and k) caused an ND panzootic in Africa, Europe, the Middle East, and Asia (2–4). In this study, we compared the full-length genomes of newly emerged strains of genotype VII NDVs to that of the currently used vaccine strain, LaSota.

Strains Chicken/Indonesia/Tangerang/004WJ/14 (Tangerang) and Chicken/Indonesia/VD/003WJ/11 (VD) were isolated from the brain samples of two chickens vaccinated against NDV with the live LaSota vaccine. The viruses were isolated from two layer farms with high mortality located in different geographical locations in West Java, Indonesia, in 2011 and 2014. The viruses were isolated by inoculating embryonated specific-pathogen-free (SPF) chicken eggs and harvesting allantoic fluid according to World Organisation for Animal Health (OIE) standard protocol (5). The pathogenicity of these strains was measured by mean death time (MDT) assay according to the Food and Agriculture Organization (FAO) manual (6). A QIAamp viral RNA minikit (Qiagen, USA) was used for RNA extraction, and the extracted RNA was submitted to the Australian Cancer Research Foundation (ACRF) for RNA sequencing. The cDNA library and sequencing were performed by the ACRF using a random hexamer approach (stranded mRNA-Seq kit; Kapa Biosystems, USA) as per the manufacturer’s recommendations. The Illumina MiSeq platform v3 was used for sequencing cDNA libraries and generated 2 × 300-nucleotide (nt) reads. After removing the adaptors and low-quality reads with Trimmomatic v0.36 software (7), Unicycler v.0.4.4 software was used for *de novo* assembly of a total of 817,686 reads for sample 1 and 764,502 reads for sample 2. The final assembled reads were visualized using Bandage (8). Contigs were compared to the nucleotide collection using NCBI BLAST, and two NDV contigs for Tangerang and VD were identified, with 46.52% and 46.49% genome GC contents, 15,096 and 15,179 nucleotide lengths, and 818-fold and 534-fold coverages, respectively. These contigs were compared to the Indonesian genotype VII strain Chicken/Sukorejo/019/10 (Sukorejo; GenBank accession number HQ697255.1) and showed similarities of 97.90% and 98.95%, respectively. In the contig of Tangerang, some contaminating sequences of *Pseudomonas* spp. were observed after an NCBI BLAST search at the end of the sequence; they were removed with BioEdit, and reverse transcriptase PCR (RT-PCR; OneStep Ahead RT-PCR, Qiagen, USA) and Sanger sequencing were used to close the detected gaps in this sequence after alignment to Sukorejo (GenBank accession number HQ697255.1). The Clustal X (9) and Genious Primer (10) software programs were used to align and annotate genes. All tools were run with default parameters.

**FIG 1:**
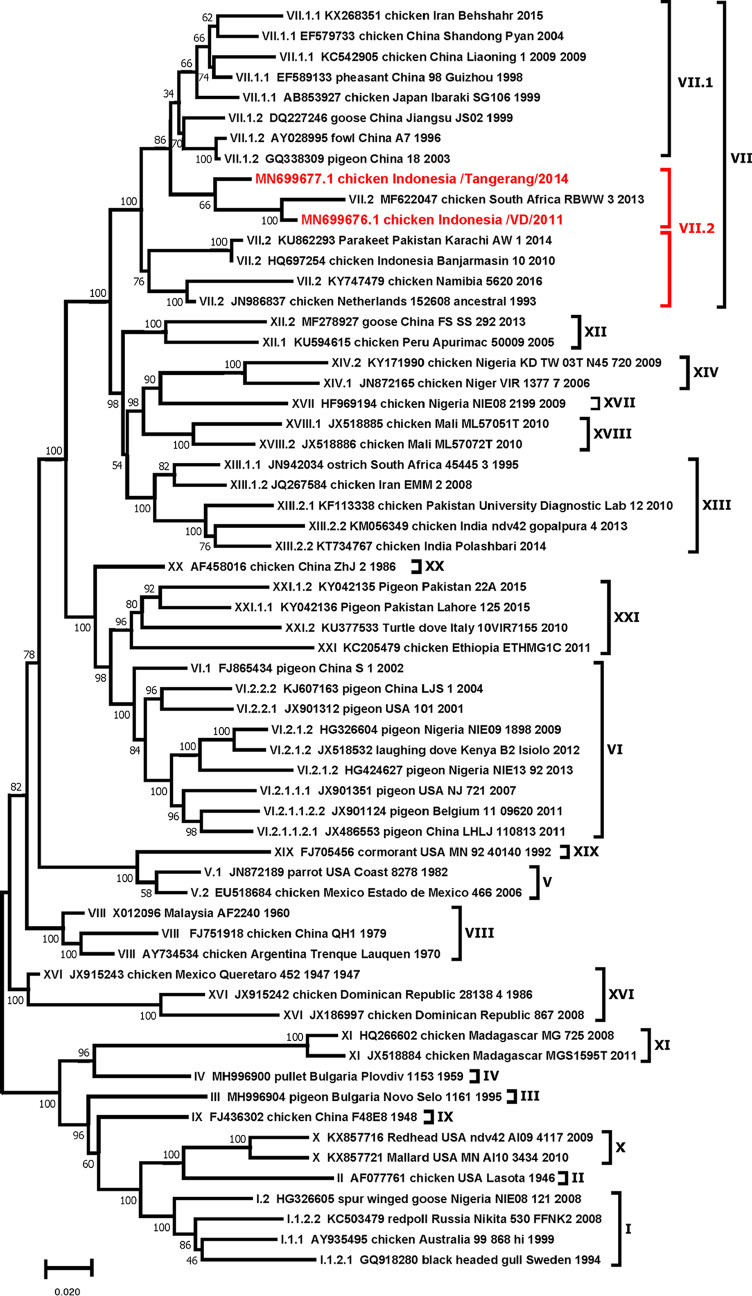
Molecular phylogenetic analysis by the maximum likelihood method. The evolutionary history was inferred by using the maximum likelihood method based on the Tamura-Nei model (14). The bootstrap consensus tree inferred from 100 replicates is taken to represent the evolutionary history of the taxa analyzed (15). Branches corresponding to partitions reproduced in less than 50% bootstrap replicates are collapsed. The percentages of replicate trees in which the associated taxa clustered together in the bootstrap test (100 replicates) are shown next to the branches (15). The initial tree(s) for the heuristic search was obtained automatically by applying the Neighbor-Join and BioNJ algorithms to a matrix of pairwise distances estimated using the maximum composite likelihood (MCL) approach and then selecting the topology with superior log likelihood value. The analysis involved 70 nucleotide sequences. All positions containing gaps and missing data were eliminated. There were a total of 326 positions in the final data set. Evolutionary analyses were conducted in MEGA7 (13). The two strains of subgenotype VII.2 highlighted in red are from this study.

Both Tangerang and VD are associated with severe neurological symptoms in infected chickens and had a mean death time (MDT) of 60 to 67 h. These two strains are similar at the C terminus of the F protein cleavage site, which is a key molecular marker for NDV pathogenicity (11, 12). The ^111^RRRKR↓F^117^ amino acid sequence motif is the same as that in Sukorejo, as the reference strain for the Indonesian genotype VII of NDVs. Phylogenetic analysis of F gene sequences carried out using MEGA7 software (13) suggests that these strains circulating in Indonesia belong to genotype VII.2 (Fig. 1), the main genotype causing recent NDV outbreaks in Indonesia. Importantly, the amino acid sequences for the viral N, P, M, F, HN, and L proteins for these two strains were similar and have percent identities of 92%, 81%, 88%, 89%, 85%, and 94% to strain LaSota (GenBank accession number AF077761.1), respectively.

These significant differences in the amino acid identities of circulation viruses and strain LaSota, as the most common vaccine strain used in Indonesia, shed more light on the probable reason for vaccine failure against these NDV strains and highlight the urgent need for updated vaccine development strategies in Southeast Asia.

### Data availability.

The GenBank accession numbers for Tangerang and VD are MN699677 and MN699676, respectively. The BioSample SRA run numbers are SRR11593162 for Tangerang and SRR11593166 for VD.

## References

[1] Rima B, Balkema-Buschmann A, Dundon WG, Duprex P, Easton A, Fouchier R, Kurath G, Lamb R, Lee B, Rota P, Wang L, ICTV Report Consortium 2019 ICTV virus taxonomy profile: Paramyxoviridae. J Gen Virol 100:1593–1594. doi:10.1099/jgv.0.001328. 31609197 PMC7273325

[2] Dimitrov KM, Abolnik C, Afonso CL, Albina E, Bahl J, Berg M, Briand F-X, Brown IH, Choi K-S, Chvala I, Diel DG, Durr PA, Ferreira HL, Fusaro A, Gil P, Goujgoulova GV, Grund C, Hicks JT, Joannis TM, Torchetti MK, Kolosov S, Lambrecht B, Lewis NS, Liu H, Liu H, McCullough S, Miller PJ, Monne I, Muller CP, Munir M, Reischak D, Sabra M, Samal SK, Servan de Almeida R, Shittu I, Snoeck CJ, Suarez DL, Van Borm S, Wang Z, Wong FYK 2019 Updated unified phylogenetic classification system and revised nomenclature for Newcastle disease virus. Infect Genet Evol 74:103917. doi:10.1016/j.meegid.2019.103917. 31200111 PMC6876278

[3] Hemmatzadeh F 2017 Molecular characterisation of newly emerged Newcastle disease viruses in Indonesia. ACIAR, Canberra, Australia.

[4] Xiao S, Paldurai A, Nayak B, Samuel A, Bharoto EE, Prajitno TY, Collins PL, Samal SK 2012 Complete genome sequences of Newcastle disease virus strains circulating in chicken populations of Indonesia. J Virol 86:5969–5970. doi:10.1128/JVI.00546-12. 22532534 PMC3347286

[5] Stear MJ 2005 OIE manual of diagnostic tests and vaccines for terrestrial animals (mammals, birds and bees) 5th Edn. Volumes 1 & 2. World Organization for Animal Health 2004. Parasitology 130:727. doi:10.1017/S0031182005007699.

[6] Grimes SE 2002 A basic laboratory manual for the small-scale production and testing of I-2 Newcastle disease vaccine. RAP publication 2002/22 FAO Regional Office for Asia and the Pacific, Bangkok, Thailand http://www.fao.org/3/AC802E/AC802E00.htm.

[7] Bolger AM, Lohse M, Usadel B 2014 Trimmomatic: a flexible trimmer for Illumina sequence data. Bioinformatics 30:2114–2120. doi:10.1093/bioinformatics/btu170. 24695404 PMC4103590

[8] Wick RR, Schultz MB, Zobel J, Holt KE 2015 Bandage: interactive visualization of de novo genome assemblies. Bioinformatics 31:3350–3352. doi:10.1093/bioinformatics/btv383. 26099265 PMC4595904

[9] Larkin MA, Blackshields G, Brown NP, Chenna R, McGettigan PA, McWilliam H, Valentin F, Wallace IM, Wilm A, Lopez R, Thompson JD, Gibson TJ, Higgins DG 2007 Clustal W and Clustal X version 2.0. Bioinformatics 23:2947–2948. doi:10.1093/bioinformatics/btm404. 17846036

[10] Kearse M, Moir R, Wilson A, Stones-Havas S, Cheung M, Sturrock S, Buxton S, Cooper A, Markowitz S, Duran C, Thierer T, Ashton B, Meintjes P, Drummond A 2012 Geneious Basic: an integrated and extendable desktop software platform for the organization and analysis of sequence data. Bioinformatics 28:1647–1649. doi:10.1093/bioinformatics/bts199. 22543367 PMC3371832

[11] Panda A, Huang Z, Elankumaran S, Rockemann DD, Samal SK 2004 Role of fusion protein cleavage site in the virulence of Newcastle disease virus. Microb Pathog 36:1–10. doi:10.1016/j.micpath.2003.07.003. 14643634 PMC7125746

[12] de Leeuw OS, Koch G, Hartog L, Ravenshorst N, Peeters BPH 2005 Virulence of Newcastle disease virus is determined by the cleavage site of the fusion protein and by both the stem region and globular head of the haemagglutinin–neuraminidase protein. J Gen Virol 86:1759–1769. doi:10.1099/vir.0.80822-0. 15914855

[13] Kumar S, Stecher G, Tamura K 2016 MEGA7: Molecular Evolutionary Genetics Analysis version 7.0 for bigger datasets. Mol Biol Evol 33:1870–1874. doi:10.1093/molbev/msw054. 27004904 PMC8210823

[14] Tamura K, Nei M 1993 Estimation of the number of nucleotide substitutions in the control region of mitochondrial DNA in humans and chimpanzees. Mol Biol Evol 10:512–526. doi:10.1093/oxfordjournals.molbev.a040023. 8336541

[15] Felsenstein J 1985 Confidence limits on phylogenies: an approach using the bootstrap. Evolution 39:783–791. doi:10.1111/j.1558-5646.1985.tb00420.x. 28561359

