# Correction for Rabiei et al., “Genome Sequences of Newly Emerged Newcastle Disease Virus Strains Isolated from Disease Outbreaks in Indonesia”

**DOI:** 10.1128/MRA.00681-20

**Published:** 2020-09-17

**Authors:** Mohammad Rabiei, Mohamad Indro Cahyono, Phuong Thi Kim Doan, Putri Pandarangga, Simson Tarigan, Risa Indriani, Indi Dharmayanti, Jagoda Ignjatovic, Wai Yee Low, Rick Tearle, Milton M. McAllister, Mohammed Alsharifi, Farhid Hemmatzadeh

**Affiliations:** a School of Animal and Veterinary Sciences, The University of Adelaide, Adelaide, Australia; b Indonesian Research Centre for Veterinary Science, Bogor, West Java, Indonesia; c School of Animal and Veterinary Sciences, Tay Nguyen University, Dak Lak, Vietnam; d Department of Veterinary Pathology, Nusa Cendana University, Kupang, Indonesia; e School of Veterinary Science, The University of Melbourne, Melbourne, Victoria, Australia; f Davies Research Centre, School of Animal and Veterinary Sciences, The University of Adelaide, Adelaide, Australia; g Research Centre for Infectious Diseases, School of Biological Sciences, The University of Adelaide, Adelaide, Australia

## AUTHOR CORRECTION

Volume 9, no. 23, e00204-20, 2020, https://doi.org/10.1128/MRA.00204-20. Page 1, abstract, line 5: “genotype VII.2” should read “genotype VII.1.”

Page 2: Figure 1 should appear as shown in this correction.

**Figure 1:**
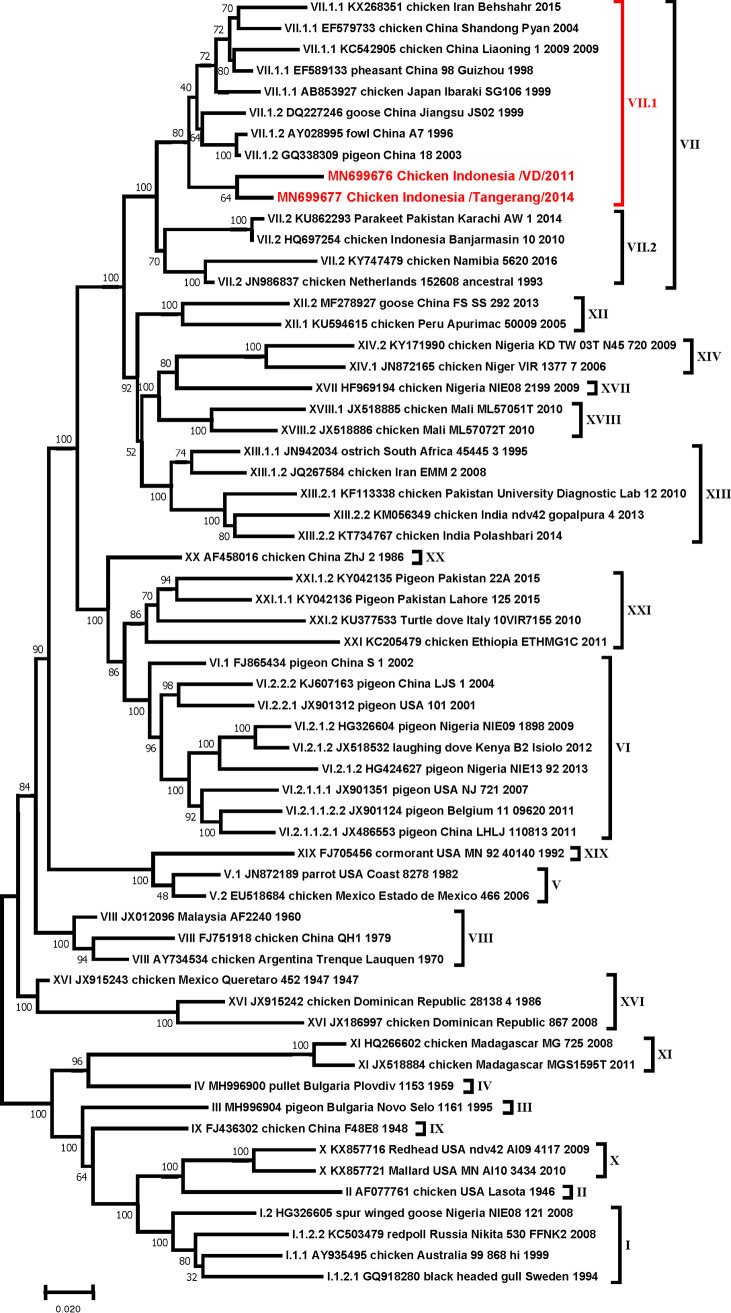


Page 3, figure legend, lines 8 and 9: “subgenotype VII.2” should read “subgenotype VII.1.”

Page 3, line 19: “genotype VII.2” should read “genotype VII.1.”

